# Modulating the Mechanical Properties of Electrospun PHB/PCL Materials by Using Different Types of Collectors and Heat Sealing

**DOI:** 10.3390/polym12030693

**Published:** 2020-03-20

**Authors:** Irena Borisova, Olya Stoilova, Nevena Manolova, Iliya Rashkov

**Affiliations:** Laboratory of Bioactive Polymers, Institute of Polymers, Bulgarian Academy of Sciences, Acad. G. Bonchev St, bl. 103A, 1113 Sofia, Bulgaria; borisova@polymer.bas.bg (I.B.); manolova@polymer.bas.bg (N.M.); rashkov@polymer.bas.bg (I.R.)

**Keywords:** polyhydroxybutyrate, polycaprolactone, dual-jet electrospinning, patterned collectors, heat sealing, mechanical properties

## Abstract

Two-component fibrous materials based on poly(3-hydroxybutyrate) (PHB, T_m_ = 160 °C) and poly(ε-caprolactone) (PCL, T_m_ = 60 °C) were successfully fabricated by dual-jet electrospinning of their separate spinning solutions. The desired alignment of the fibers that compose PHB/PCL mats was achieved by using three types of rotating collectors—drum (smooth), blade and grid. Additional fiber alignment in the direction of collector rotation was achieved by rotating at 2200 rpm. Moreover, the selected concentration of PCL spinning solution resulted in fibers with spindle-like defects along their length. Thus, “segment” sealing of the PHB (high-melting) fibers by the molten PCL (low-melting) fibers/defects sites was achieved after heating the PHB/PCL mats above the melting temperature (T_m_) of PCL. The surface morphology, thermal behavior and mechanical properties of the PHB/PCL mats before and after thermal treatment were characterized by scanning electron microscopy (SEM), Fourier transform infrared (FT-IR) spectroscopy, differential scanning calorimetry (DSC) and mechanical tests. The results indicated that regardless of the cutting direction of the specimens (0° or 90°), thermal treated PHB/PCL mats reveal enhanced mechanical properties. Therefore, this work provides an easily feasible route for the fabrication of electrospun PHB/PCL mats with tunable mechanical properties and improved performance.

## 1. Introduction

Electrospinning is a cutting-edge nanotechnology and currently the only technique that allows facile fabrication of non-woven textiles (the so-called “mats”) with unique features and versatile possible applications [1,2,3,4]. However, despite the progress gained in terms of the electrospinning process, finding possibilities for tuning and improving the mechanical properties of the mats still remains a major challenge.

The mechanical properties of the electrospun mats depend on diverse parameters. A key factor is the structure of the non-woven textile; mats composed of aligned fibers are characterized by better mechanical behavior compared to mats composed of randomly deposited fibers [5,6,7,8,9]. Therefore, the achievement of a certain alignment of the fibers that compose the mats is a suitable approach for improving the strength of the mats. Another approach is the thermal treatment of the mats at temperatures close to or above their melting temperatures (T_m_). Finding the conditions at which thermal sealing is achieved while preserving the fibrous structure of the mat still remains a challenge. There are very few reports on the improvement of the mechanical behavior of mats by thermal treatment [10,11,12,13]. From the aforementioned, it is clear that the innovative combination of electrospinning of suitably selected polymers on collectors with specific patterns that may enable fibers alignment and controlled mats structure followed by thermal treatment of the mats, is an elegant solution that can lead to the preparation of mats with improved mechanical behavior.

Poly(3-hydroxybutyrate) (PHB) deserves special interest—it is recognized as the prototypical biodegradable thermoplastic to solve the waste disposal challenge [14]. Because of its high crystallinity, PHB is stiff and brittle, and this results in very poor mechanical properties with a low extension at break, which limits its range of applications [15]. Numerous reports are devoted to the preparation of materials by solvent casting or compression molding of PHB blends with poly(ε-caprolactone) (PCL) [16,17,18,19]. The electrospinning of mixed PHB/PCL solutions has been reported [20,21]. However, there are still no reports on the simultaneous electrospinning of separate PHB and PCL spinning solutions. Recently, we have shown that the electrospinning of a poly(L-lactic acid) (PLA) in conjunction with the electrospraying of PCL results in the fabrication of PLA mats decorated with PCL particles [13]. After thermal treatment at T_m_ of PCL (60 °C), mats were characterized by enhanced mechanical properties, because of the “spot” sealing of the PLA fibers in the area of the molten PCL particles.

In this respect, the focus of the present study is on the fabrication of two-component mats from PHB (high-melting) and PCL (low-melting) by the dual-jet electrospinning of their separate spinning solutions and subsequent thermal treatment at a temperature above the melting temperature of PCL. In order to tune the mechanical properties of the fabricated PHB/PCL mats, both the concentration of the PCL spinning solution used and heat treatment conditions were varied and the optimal parameters were found. Moreover, collectors with specific patterns (blade and grid) that may enable additional alignment of the fibers were applied, as well as a collector rotation speed of 2200 rpm. The detailed morphology and thermal behavior of the PHB/PCL mats before and after thermal treatment were studied by SEM and DSC, respectively. The effect of the thermal treatment on the mechanical properties of the PHB/PCL mats was assessed by tensile tests. In addition, the effect of the direction of specimen cutting (0° or 90°) on the mechanical properties was also studied with respect to the collector rotation direction. Finally, the combination of dual-jet electrospinning of PHB and PCL with subsequent thermal treatment—enabling the “segment” sealing of the PHB fibers—is a valuable strategy for enhancing the mechanical properties of the PHB/PCL mats.

## 2. Materials and Methods

### 2.1. Materials

Poly(3-hydroxybutyrate) (PHB, 330000 g/mol, Biomer) and poly(ε-caprolactone) (PCL, 80000 g/mol, Capa™6800, Perstorp Holding AB) were used. Chloroform (CHCl_3_, ≥ 99.8%), dichloromethane (DCM, ≥ 99.8%) and *N,N*-dimethylformamide (DMF, ≥ 99.8%) were supplied by Merck. All chemicals were of analytical grade and were used without further purification.

### 2.2. Dual-Jet Electrospinning

**Figure 1:**
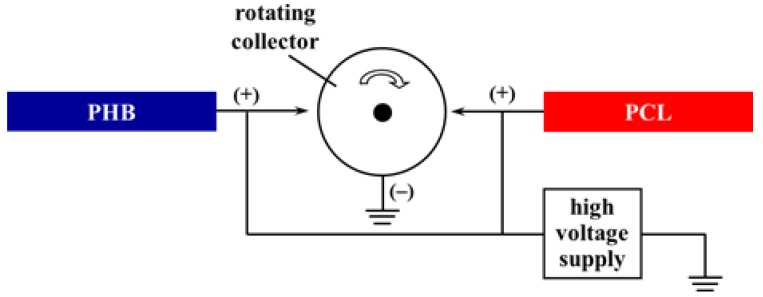
Scheme of the experimental dual-jet electrospinning set-up.

For the preparation of all types of mats, PHB spinning solutions (14% w/v) in CHCl_3_/DMF (4/1) mixed solvent were prepared by heating at 60 °C using a reflux condenser. PCL was dissolved in DCM/DMF (9/1) to form the PCL spinning solution (10% w/v). The prepared solutions were loaded into two separate plastic syringes (10 mL) placed horizontally in two syringe pumps (NE-300, New Era Pump Systems, Inc.) and were delivered at a constant feed rate of 3 mL/h. These pumps were positioned at an angle of 180° in respect to the rotating collector (Figure 1). The tip-to-collector distance was 25 cm for the electrospinning of the PHB solution and 15 cm for the PCL solution. The electrospinning was conducted at a constant applied voltage of 25 kV by using a common high-voltage power supply and at a constant collector rotation speed of 2200 rpm. Finally, the collected PHB/PCL mats were dried under a reduced pressure at 30 °C to remove any residual solvents.

Three types of PHB/PCL mats were prepared by dual-jet electrospinning of separate PHB (5 mL) and PCL (5 mL) solutions onto three types of rotating collectors—drum (smooth), blade and grid (patterned). The blade collector consisted of 18 parallel steel blades spaced 11 mm from each other. The grid one consisted of a steel grid of 2 mm thickness and a rhomboid (10 × 10 mm) pattern and was mounted on a wooden drum collector. For clarity, the prepared mats will be denoted to as follows: PHB/PCL (drum), PHB/PCL (blade) and PHB/PCL (grid).

### 2.3. Thermal Treatment of the PHB/PCL Mats

The three prepared types of PHB/PCL mats were heat treated in a vacuum drying chamber (Binder, VD 23) at 80 °C for 15 min. In order to prevent any shrinking of the materials during the treatment, rectangular specimens with 200 mm × 160 mm dimensions were cut and fixed in a stainless steel appliance fitted with pressed clamps.

### 2.4. Characterization of the PHB/PCL Mats

The detailed morphology of the PHB/PCL mats before and after the thermal treatment was observed by SEM. Specimens were placed on the sample holders, vacuum-coated with gold in a Jeol JFC-1200 fine coater and analyzed using Jeol JSM-5510 or Philips 515 instruments. The mean fiber diameter, the characteristics of the spindle-like defects (mean length and mean width) and the standard deviation were determined by measuring at least 15 fibers from SEM micrographs using Image J software [22]. 

Attenuated total reflection Fourier transform infrared spectroscopic (FTIR-ATR) analysis was performed using an IRAffinity-1 spectrophotometer (Shimadzu Co., Kyoto, Japan) equipped with a MIRacle™ ATR (diamond crystal, depth of penetration of the IR beam into the sample was about 2 µm) accessory (PIKE Technologies, Madison, WI, USA). The spectra were recorded in the range of 4000–600 cm^−1^ with a spectral resolution of 4 cm^−1^ using a DLATGS detector equipped with a temperature controller. All spectra were corrected for H_2_O and CO_2_ using IRsolution internal software (Shimadzu Co., Kyoto, Japan). All samples were dried under reduced pressure prior to analysis.

Differential scanning calorimetry (DSC) was performed on DSC Q200 equipment (TA Instruments, New Castle, DE, USA) in the temperature range of –50 °C to 200 °C with a heating rate of 10 °C/min and under a nitrogen flow. The melting temperatures (T_m_) were obtained from the DSC endotherms (first heating run).

### 2.5. Mechanical Testing of the PHB/PCL Mats

The mechanical properties of the PHB/PCL mats before and after the thermal treatment were studied by tensile test measurements on a single column dynamometer INSTRON 3344 equipped with a 50 N load cell. Rectangular specimens with 20 mm width and 60 mm length were cut and used for analysis at a crosshead speed of 20 mm/min at room temperature. The specimens were cut in the collector rotation direction (0°, parallel) and perpendicular to the collector rotation direction (90°). The thickness of each specimen was measured before the tensile test using a Digital Thickness Gauge FD 50 (Käfer GmbH, Germany) and was ca. 250 µm. The test was carried out in accordance with the ASTM D-638 procedure. The mechanical characteristics—average values of the Young’s modulus (E, MPa), tensile strength (σ, MPa) and elongation at break (ε_B_, %)—were determined based on the regression of the linear part of the stress–strain curves from at least ten tested specimens of each material.

## 3. Results and Discussion

### 3.1. Fabrication of PHB/PCL Mats

**Figure 2:**
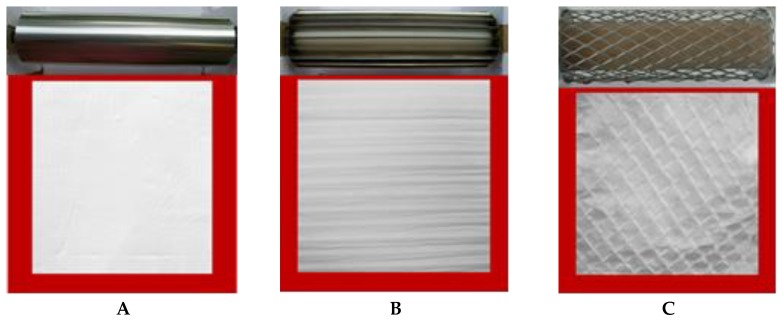
Digital photos of the rotating collectors used—drum (**A**), blade (**B**) and grid (**C**)—and photos of the fabricated PHB/PCL mats, respectively.

In the present study, three types of mats based on PHB (high-melting) and PCL (low-melting) were fabricated by dual-jet electrospinning onto three types of rotating collectors—drum (smooth), blade and grid (patterned) (Figure 2).

The use of the rotating drum (Figure 2A) collector provided excellent efficiency of deposition of the fibers. However, the degree of alignment was far below expected. For this reason, the drum collector was additionally modified as the rotating “blade” (Figure 2B) and “grid” (Figure 2C). In these cases, highly aligned fibers in the gap between the blades and in grid holes could be collected. In that manner—modifying the jet movement by controlling the electric field distribution—the targeted design of the fabricated mats was obtained. In order to achieve the desired alignment of the fibers, even in the case of the rotating drum collector, the speed was fixed at 2200 rpm.

By varying the conditions for dual-jet electrospinning (voltage, tip-to-collector distance and feeding rates of the spinning solutions), the optimal conditions for the formation of a Taylor cone were found for both PHB and PCL spinning solutions. In all experiments, equal volumes (5 mL) of PHB and PCL spinning solutions were used. This allowed the fabrication of mats with customized designs and appropriate thicknesses for tensile tests (~250 µm). Moreover, the concentration of the PCL solution was purposively selected to result in the formation of spindle-like defects along the fibers’ lengths during the electrospinning.

In order to find the temperature and time at which “segment” sealing of the fibers is achieved while preserving the fibrous structure of the mats, preliminary experiments were performed. Three different temperatures (60, 70 and 80 °C), which are close to the melting temperature of PCL (T_m_ = 60 °C) and three different heating times—10, 15 and 20 min—were selected. It was found that by heating at 80 °C for 15 min, the desired melting of the PCL defects/fibers was achieved while preserving the fibrous structure.

**Figure 3:**
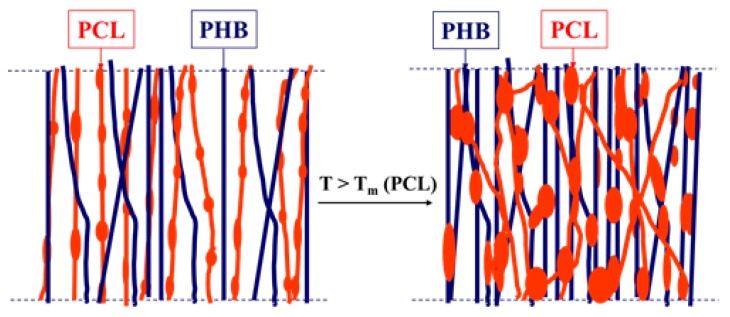
Schematic representation of the working hypothesis.

The working hypothesis is shown in Figure 3. Thus, by innovative combining of different methods, a series of PHB/PCL mats with tailored design, structure and properties could be obtained.

### 3.2. Effect of Thermal Treatment on the Morphology of the PHB/PCL Mats

**Figure 4:**
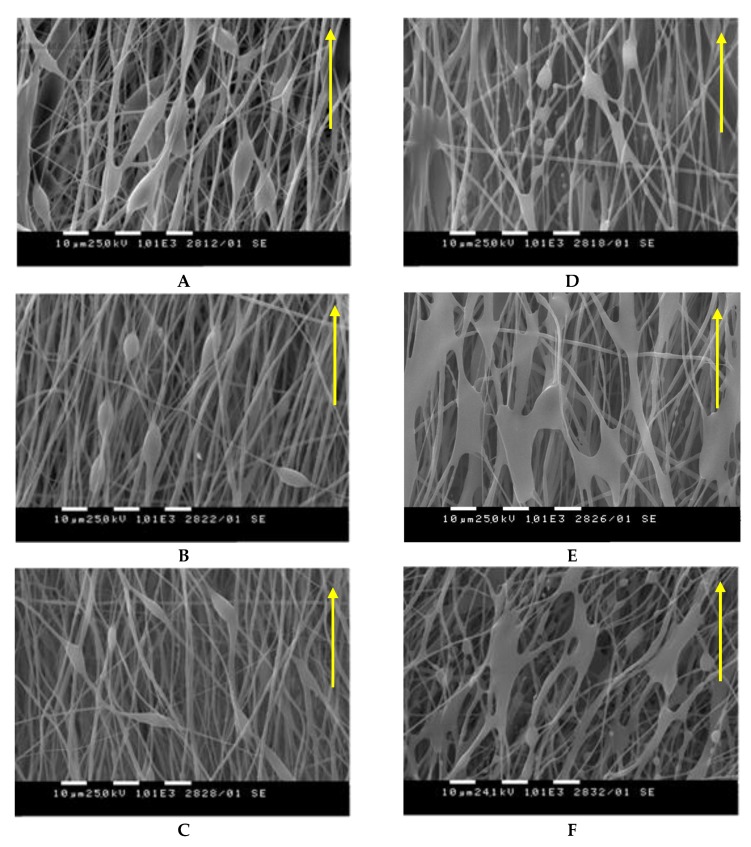
SEM micrographs of the PHB/PCL mats before (**A–C**) and after (**D–F**) thermal treatment onto rotating drum (**A,D**), blade (**B,E**) and grid (**C,F**) collectors. The direction of the collector rotation is indicated by arrows.

Changes in the morphology of the obtained PHB/PCL mats before and after thermal treatment were observed by SEM (Figure 4). It is evident that in all cases, the selected rotation speed (2200 rpm) resulted in the formation of fibers with a certain alignment along the direction of the collector rotation. This tendency also persisted after the thermal treatment of the mats.

It is noteworthy that, depending on the concentration of the spinning solutions, which were subjected to dual-jet electrospinning, the morphology of the fibers was different. From the presented SEM micrographs, it can be seen that before thermal treatment (Figure 4A–C), the dual-jet electrospinning of PHB and PCL under the selected conditions and concentrations resulted in the formation of two types of fibers—defect-free cylindrical ones and fibers with spindle-like defects along their length. The average fiber diameters of the defect-free fibers before thermal treatment were 907 ± 94 nm for PHB/PCL (drum), 1072 ± 103 nm for PHB/PCL (blade) and 1084 ± 98 nm for PHB/PCL (grid), whereas those of the fibers with spindle-like defects were 586 ± 86 nm, 794 ± 77 nm and 776 ± 81 nm, respectively. The defects have the following mean length and mean width of the “spindles” (L/W): 20 ± 6.1 µm/6.3 ± 0.8 µm for PHB/PCL (drum), 15 ± 2 µm/5.9 ± 0.2 µm for PHB/PCL (blade) and 16.7 ± 2.3 µm/4.0 ± 0.7 µm for PHB/PCL (grid).

As seen from SEM micrographs (Figure 4D–F), in general, the fibrous structure of the PHB/PCL mats was preserved after thermal treatment above the T_m_ of PCL. However, “segment” sealing of the fibers in the molten sites along their length was achieved. Probably because of the heat treatment above the T_m_, the formation of “segment” sealing was observed only in the molten PCL defect sites. Therefore, it is evident that the defect-free fibers were of PHB, while those with spindle-like defects were of PCL. After thermal treatment, the mean fiber diameters were 1031 ± 135 nm for PHB/PCL (drum), 1341 ± 404 nm for PHB/PCL (blade) and 1120 ± 268 nm for PHB/PCL (grid). This slight increase in the mean diameters was probably due to the covering of the PHB fibers by the molten PCL.

**Figure 5:**
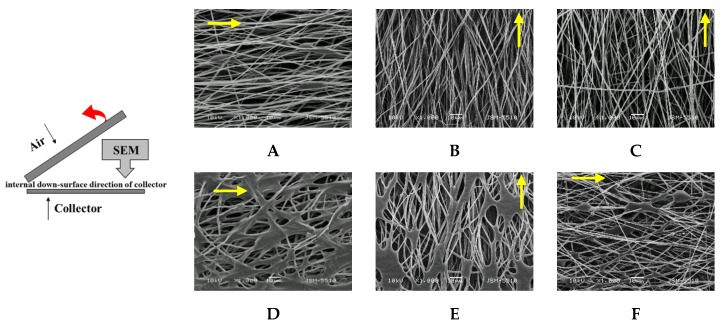
SEM micrographs of the internal surface in direction to the collector of the PHB/PCL mats before (**A–C**) and after (**D–F**) thermal treatment onto rotating drum (**A,D**), blade (**B,E**) and grid **(C,F**) collectors. The direction of the collector rotation is indicated by arrows.

To prove that the morphology of the PHB/PCL mats and their fiber alignment were preserved in the bulk of the mats, their internal structure was also observed by SEM (Figure 5). For this purpose, the PHB/PCL mats were longitudinally cleaved—as shown in the scheme in Figure 5—and the inner morphology that was in contact with the collector, before and after heat treatment, was analyzed. Again, two types of fibers—defect-free cylindrical ones and fibers with spindle-like defects along their length (Figure 5A–C)—are clearly seen. Since this is the inner surface of the mats, most of the defects are seen in depth. Apparently, the heating leads to melting of the PCL, “segment” sealing and interconnectivity of the fibers (Figure 5D–F). Obviously, like the surface morphology, the bulk morphology of the PHB/PCL mats remained unchanged even after the heat treatment.

The obtained SEM results were in agreement with the hypothesis presented in Figure 3 and showed that suitable conditions for the fabrication of two-component PHB/PCL mats with well-defined morphology (on the surface and in the bulk) and fiber alignment in the direction of the collector rotation were developed. The dual-jet electrospinning provided a uniform distribution of the PHB and PCL fibers throughout the mats. Moreover, the appropriate conditions for a thermal treatment that preserves the fibrous structure and causes segment “sealing” of the fibers were also found.

### 3.3. Characteristics of the PHB/PCL Mats

#### 3.3.1. FT-IR Analysis

**Figure 6:**
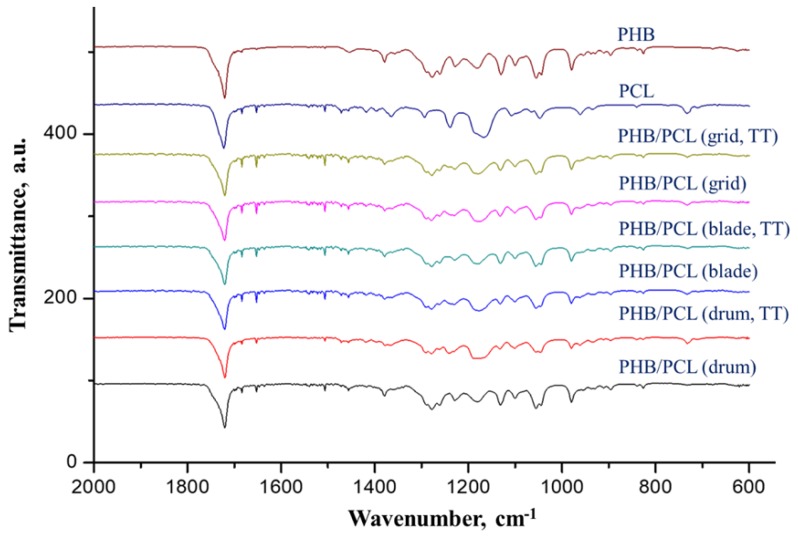
FTIR-ATR spectra of the PHB/PCL mats prepared onto rotating drum, blade and grid collectors before and after thermal treatment (TT).

In order to prove that the dual-jet electrospinning conditions led to the uniform distribution of PHB and PCL fibers in the mats, FTIR-ATR analysis was performed. The spectra of the PHB/PCL mats were compared with the spectra of neat PHB and neat PCL mats (Figure 6).

The IR spectrum of the PHB mats showed the following characteristic bands: at 1720 cm^−1^ with a narrow shoulder at 1735 cm^−1^ (C=O stretching vibrations); 1456 cm^−1^ (–CH_3_ asymmetric deformation); 1379 cm^−1^ (–CH_3_ symmetric deformation); 1276 cm^−1^, 1228 cm^−1^ and 1180 cm^−1^ (C–O–C stretching); 1261 cm^−1^ (C–O–C stretching and CH deformation); 1055 cm^−1^ (C–O stretching); and 1043 cm^−1^ (C–CH_3_ stretching). The bands were in accordance with the literature [23].

The main bands in the spectrum of the PCL mats were as follows: at 1722 cm^−1^ (C=O stretching vibrations); at 1471 cm^−1^, 1417 cm^−1^ and 1363 cm^−1^ (–CH_2_ bending vibrations); at 1240 cm^−1^ and 1165 cm^−1^ (C–O–C stretching); and at 1107 cm^−1^ and 1047 cm^−1^ (C–O stretching) [24].

Characteristic bands for both the PHB and PCL were observed in the spectrum of the PHB/PCL mats before and after thermal treatment. A strong band for ester groups appeared at 1722 cm^−1^. In addition, bands were also observed at 1471 cm^−1^ and 1363 cm^−1^ for PCL (–CH_2_ bending vibrations), as well as at 1456 cm^−1^ and 1379 cm^−1^ for PHB (–CH_3_ asymmetric and symmetric deformation).

The results showed that the prepared mats consisted of both PHB and PCL fibers.

#### 3.3.2. DSC Analysis

**Figure 7:**
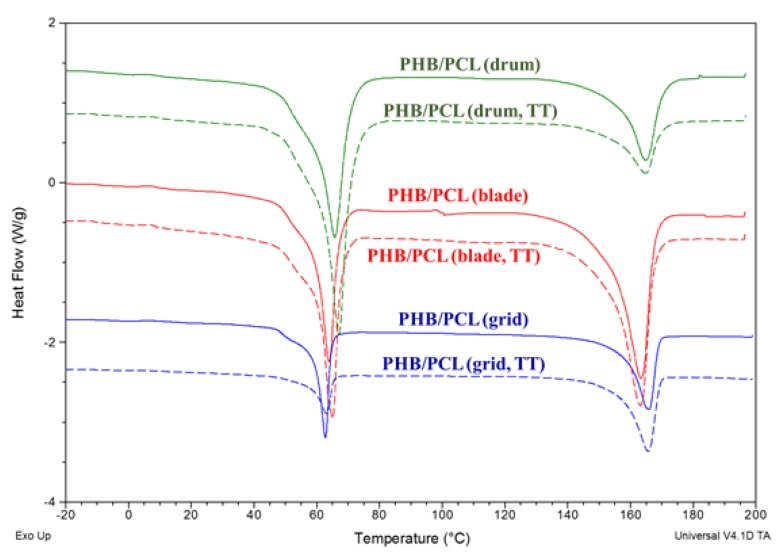
DSC thermograms (first heating run) of the PHB/PCL mats before (solid lines) and after (dash lines) thermal treatment onto rotating drum (green), blade (red) and grid (blue) collectors.

The thermal behavior of the obtained PHB/PCL mats was studied using DSC measurements. As shown in Figure 7, two distinct endothermic peaks at the intervals 63–67 °C and 163–166 °C, corresponding to the melting temperatures of the PCL and PHB respectively, were observed. These results were expected, because fibers from PHB and PCL are deposited separately by dual-jet electrospinning. Therefore, the melting process was weakly affected by the presence of two different polymers, indicating immiscibility between them.

However, the peaks for the T_m_ of PCL before and after thermal treatment were shifted to lower temperatures when using the rotating blade and grid collectors in comparison with the drum. The difference in the thermal behavior of the PHB and PCL fibers in the mats was probably due to the fact that PHB is a high crystallinity polymer, whereas PCL is a semi-crystalline ductile polymer [25]. On the other hand, the processes of electrospinning and thermal treatment at temperatures higher than the T_m_ of PCL had an additional influence on the thermal behavior of the PHB/PCL mats [26]. It can be assumed that during the thermal treatment, the PHB probably played the role of the nucleating agent for the PCL. In addition, the alignment of the fibers in the PHB/PCL mats facilitated their crystallization and led to the melting of the PCL fibers at lower temperatures.

### 3.4. Effect of Thermal Treatment on the Mechanical Properties of the PHB/PCL Mats

**Figure 8:**
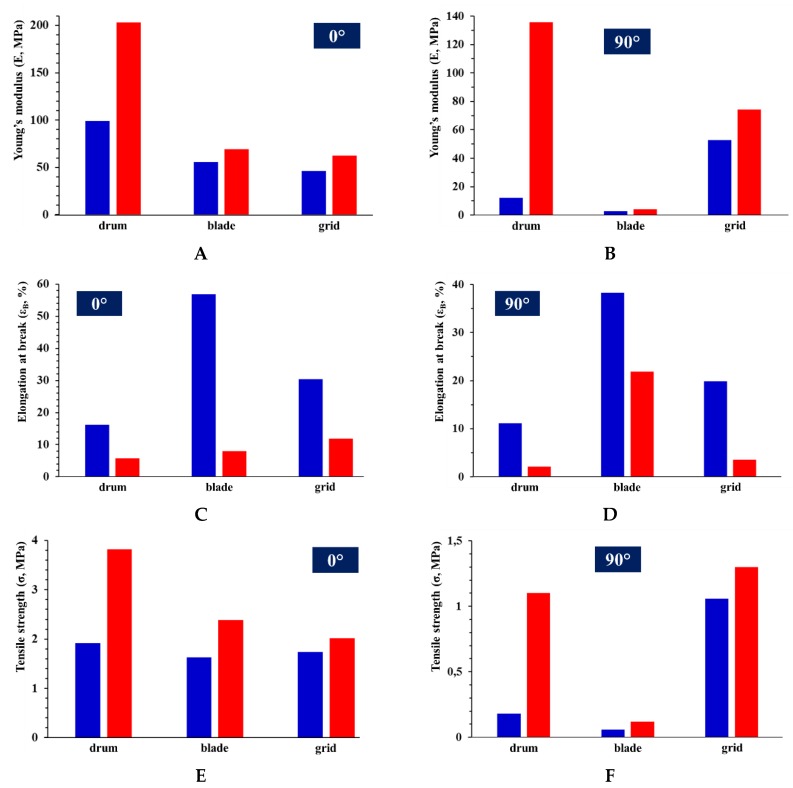
Modulus of elasticity (**A,B**), elongation at break (**C,D**) and tensile strength (**E,F**) of the PHB/PCL mats before (blue) and after (red) thermal treatment onto three types of rotating collectors and at different directions of cutting (0° or 90°).

The mechanical properties of electrospun fibrous materials depend on diverse parameters. A problem with studying the mechanical properties of electrospun materials is the lack of specific standardization for conducting tensile tests. One of the important factors that has great significance to the mechanical properties of the mats is the direction in which the samples from a non-woven textile have to be cut: parallel (0°) or at an angle (90° or others) to the collector rotation direction. Recently, we have reported that for samples cut in the direction of the collector rotation, the probability that a greater number of fibers will be gripped between the grips of the tensile testing apparatus is higher compared to samples cut in at an angle (90° or others) to the collector rotation [13]. Thus, these cases show better mechanical parameters. In order to confirm these findings, tensile tests for the PHB/PCL mats were performed with specimens cut at an angle of 0° or 90° to the collector rotation direction. Based on the obtained stress–strain curves (Figure S1), the values of the modulus of elasticity (E, MPa), tensile strength (σ, MPa) and the elongation at break (ɛ_B_, %) were determined (Figure 8).

In general, regardless of the cutting direction of the specimens (0° or 90°) and collector type, thermal-treated PHB/PCL revealed enhanced tensile strength. Apparently, heating the PHB/PCL mats resulted in the PCL melting, leading to “segment” sealing and interconnectivity of the fibers in the molten sites, and thus to increasing the strength of the PHB/PCL mats. It should be mentioned that the thermal heating had a slight effect on the mechanical properties of the PHB/PCL (drum) mats cut at 90° compared to the patterned PHB/PCL (blade) and PHB/PCL (grid). This is probably due to the lack of additional alignment of the fibers when using a collector of such geometry.

Before thermal treatment, regardless of the cutting direction of the samples (0° or 90°), all three types of mats were characterized by mechanical properties characteristic of PCL—low modulus and tensile strength and a significant increase in the elongation at break.

Thermal treatment of the materials resulted in a significant increase in the modulus of elasticity and tensile strength, and a decrease in elongation at break. Moreover, the thermal treatment of the PHB/PCL mats resulted in greater values of the modulus of elasticity and tensile strength for samples cut in the 0° direction than in the 90°. In contrast, the values of the elongation at break decreased after thermal treatment. Probably, the thermal treatment reduces the effect of the PCL because of its melting and the elongation at break of PHB becomes prevailing in PHB/PCL mats.

It should be noted that all samples cut in the 90° direction were characterized by lower values of the modulus of elasticity and tensile strength compared to those cut in the 0° direction. This further confirms that, for samples cut in the 0° direction, a greater number of fibers are gripped between the grips of the testing apparatus as compared to samples cut in the 90° direction.

These results further demonstrate that, PCL fibers improve the elasticity and reduce the brittleness of thermally treated PHB/PCL mats cut in the 0° direction. Heat treatment of the PHB/PCL mats above the T_m_ of PCL results in enhanced mechanical properties, regardless of the cutting direction (0° or 90°) and collector type.

## 4. Conclusions

For the first time, two-component PHB/PCL mats were fabricated by dual-jet electrospinning of their separate spinning solutions. Moreover, for the first time, by electrospinning onto three types of rotating collectors (drum, blade and grid) at 2200 rpm, targeted design of the fabricated mats was obtained ensuring the fibers’ alignment in the direction of collector rotation. In that way, one of the prerequisites for improving the mechanical properties of the mats was achieved. The obtained results indicate that the selected concentration of the PCL spinning solution results in PCL fibers with spindle-like defects along their length, which was a desirable effect in terms of thermal treatment. Furthermore, thermal treatment near the melting temperature of PCL enabled sealing of the fibers while preserving the fibrous structure, thus enhancing the mechanical properties of the mats. Furthermore, samples cut in the direction of 0° show better mechanical properties than those cut in the direction of 90°. The proposed approach is an original, easy and feasible strategy for tuning the mechanical properties of electrospun mats.

## Supplementary Materials

The following are available online at https://www.mdpi.com/2073-4360/12/3/693/s1, Figure S1: Stress–strain curves of the PHB/PCL mats before and after thermal treatment.
