# Supplementary material for: Modulating the Mechanical Properties of Electrospun PHB/PCL Materials by Using Different Types of Collectors and Heat Sealing
Source: Polymers (Basel). 2020 Mar 20;12(3):693. doi: 10.3390/polym12030693 (PMC7183258; doi:10.3390/polym12030693)
Supplement: Supplementary file 1 [file polymers-12-00693-s001.pdf]

polymers

MDPI

Supplementary data

Modulating the Mechanical Properties of Electrospun PHB/PCL Materials by Using Different Types of Collectors and Heat Sealing

Irena Borisova, Olya Stoilova*, Nevena Manolova and Iliya Rashkov

Laboratory of Bioactive Polymers, Institute of Polymers, Bulgarian Academy of Sciences, Acad. G. Bonchev St, bl. 103A, 1113 Sofia, Bulgaria; stoilova@polymer.bas.bg

* Correspondence: stoilova@polymer.bas.bg (O.S.)

The stress–strain curves shown in Figure S1 represent the behavior of PHB/PCL materials subjected to a constant load.

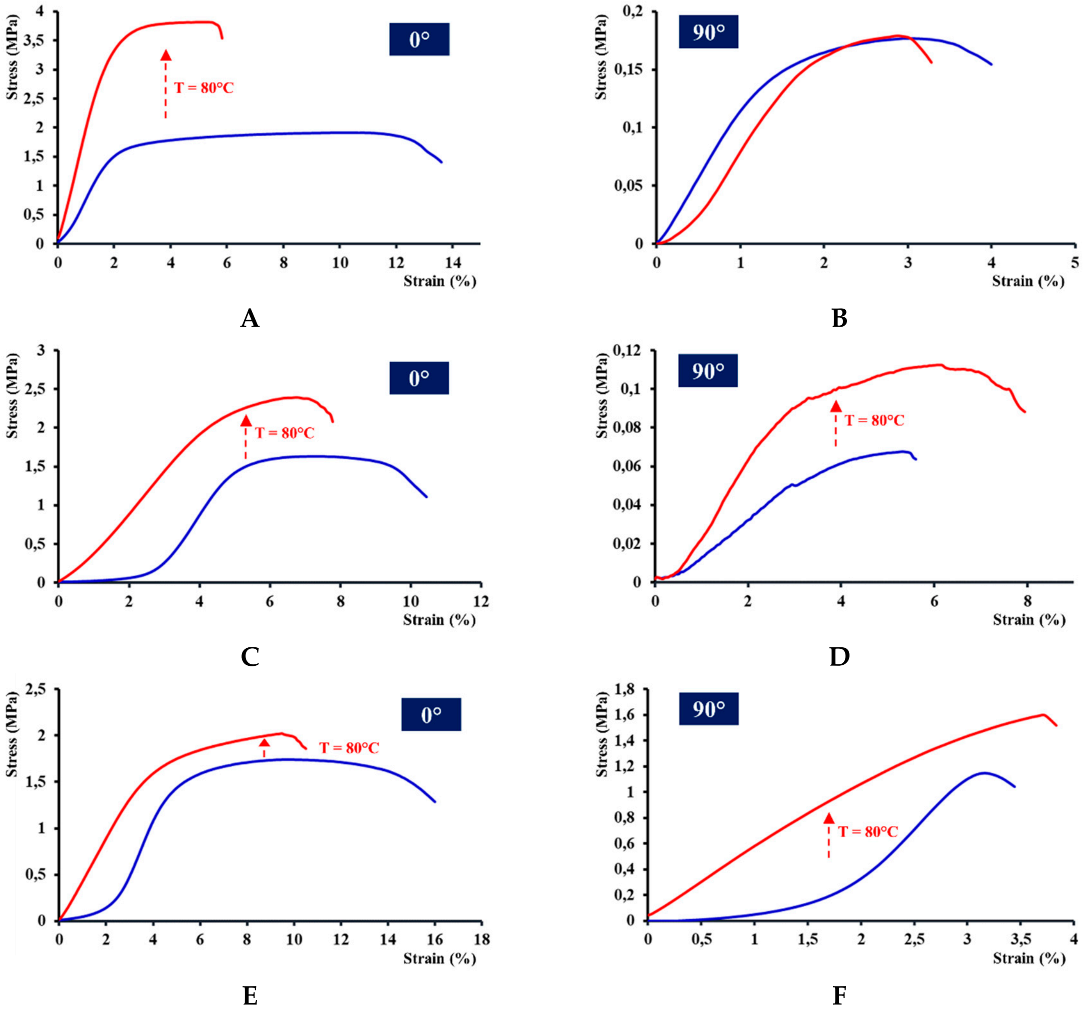

Figure S1. Stress–strain curves of the PHB/PCL mats before (blue) and after (red) thermal treatment onto rotating drum (**A, B**), blade (**C, D**) and grid (**E, F**) collectors at different directions of cutting.
